# Berberine-gut microbiota interactions based on CiteSpace: current research status and hotspots

**DOI:** 10.3389/fmicb.2026.1751887

**Published:** 2026-03-16

**Authors:** Yan Chen, Tao Chen, Shiyong Tian, Ling Tao, Fangfang Fan, Yuanyong Yang, Xiangchun Shen

**Affiliations:** 1 The State Key Laboratory of Functions and Applications of Medicinal Plants, School of Pharmaceutical Sciences, Guizhou Medical University, Guiyang, Guizhou, China; 2 The Department of Pharmacology of Materia Medica (The High Efficacy Application of Natural Medicinal Resources Engineering Center of Guizhou Province and The High Educational Key Laboratory of Guizhou Province for Natural Medicinal Pharmacology and Druggability), School of Pharmaceutical Sciences, Guizhou Medical University, Guiyang, Guizhou, China; 3 Department of Pharmacology, School of Basic Medical Sciences, Guizhou Medical University, Guiyang, Guizhou, China; 4 Department of Cardiology, The Affiliated Hospital of Guizhou Medical University, Guiyang, Guizhou, China

**Keywords:** berberine, bibliometrics, fecal microbiota transplantation, gut microbiota, metabolic-immune mechanisms, neuro-microbial crosstalk

## Abstract

**Background:**

The gut microbiota modulates host metabolic and immune homeostasis through host-microbiota interactions and microbial metabolites. Berberine (BBR), the primary active constituent of *Coptis chinensis*, has been shown to ameliorate host metabolic disorders by remodeling the gut microbial community. However, systematic reviews remain relatively scarce regarding the mechanisms underlying BBR-gut microbiota interactions.

**Methods:**

Therefore, we conducted a bibliometric analysis of 426 articles retrieved from the Web of Science Core Collection (WOSCC) and the China National Knowledge Infrastructure (CNKI) database (January 1, 2005–January 31, 2025) using CiteSpace. Analyses included publication trends, country/author collaboration networks, keyword co-occurrence and burst detection, and document co-citation analysis.

**Results:**

The results revealed a steady increase in annual publications, with China contributing the majority of studies. Author collaboration networks indicated limited integration among research groups. Keyword analysis identified key research clusters such as diabetes, inflammation, bile acid metabolism, and colorectal cancer. Chinese studies placed greater emphasis on disease applications, whereas English-language articles tended to focus on mechanistic insights. Emerging research hotspots include depression, fecal microbiota transplantation, bile acids, and ulcerative colitis. Co-citation analysis highlighted two foundational themes: microbial metabolites and metabolic-immune crosstalk.

**Discussion:**

This bibliometric study systematically outlines the research landscape of berberine-gut microbiota interactions, highlighting emerging frontiers such as neuro-microbial crosstalk, fecal microbiota transplantation (FMT) -based combination therapies, and metabolic-immune mechanisms. The findings provide valuable references for identifying research trends, fostering collaboration, and guiding future investigations in this field.

## Graphical abstract

**Figure:**
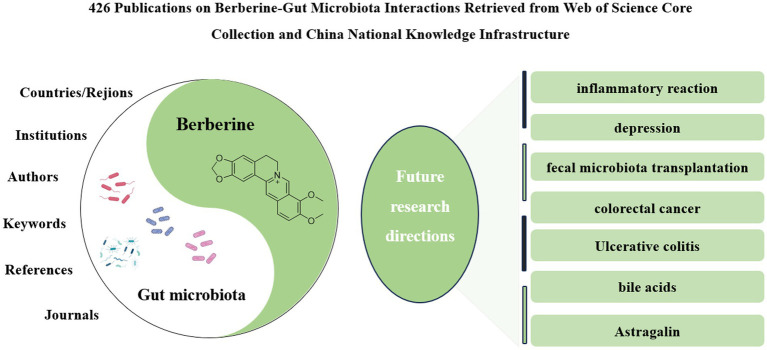

## Highlights

- Integrated dual-database (WOSCC/CNKI) analysis reveals divergent scholarly focus on berberine-gut microbiota interactions: Chinese literature prioritizes disease applications, while English studies probe deeper mechanisms, offering a seminal perspective for cross-cultural academic collaboration.
- Gut microbiota metabolites (SCFAs/bile acids) & axes (gut-liver/brain) are identified as central hubs mediating the pharmacological effects of berberine, which concurrently provide structural evidence for its modulation of metabolism-immunity crosstalk mechanisms.
- Berberine-baicalin synergy shows promise beyond hyperlipidemia, concurrently, emerging microbiome-targeted approaches (e.g., FMT and precision editing of bile acid-metabolizing microbiota) represent promising strategies to optimize therapeutic outcomes.
- Co-citation networks confirm berberine modulates “microbiota-metabolism-immunity” via metabolites (SCFAs/bile acids/TMAO), targeting metabolic disorders, colitis, and Colorectal Cancer.
- Personalized strategies integrating multi-omics address microbiome heterogeneity for precision berberine efficacy.

## 1 Introduction

The gut microbiota, functioning as a quasi-second genome, profoundly modulates energy metabolism, immune homeostasis, and neuroendocrine functions through host–microbe interactions and metabolites (e.g., SCFAs, bile acids). Its dysbiosis is mechanistically linked to metabolic disorders, immune dysregulation, and neurodegenerative diseases (Lynch and Pedersen, 2016; Fava et al., 2019; Paolella et al., 2014). Recent evidence indicates that gut microbiota disruption compromises intestinal barrier integrity, triggering systemic inflammation and metabolic endotoxemia that drive pathological progression in obesity, diabetes, cardiovascular diseases, and psychiatric conditions (Afzaal et al., 2022). Consequently, targeted microbiota modulation represents an emerging therapeutic strategy for chronic diseases.

**Figure 1:**
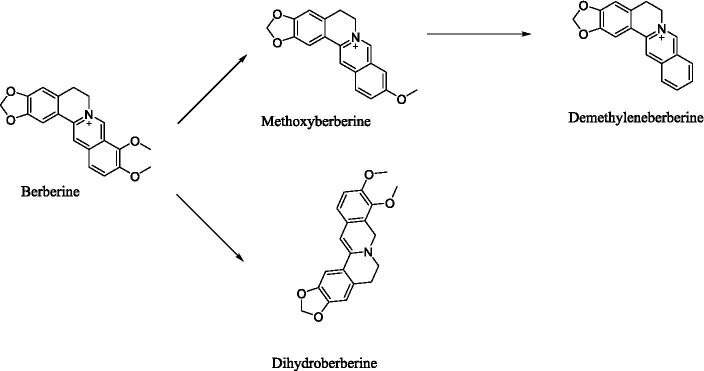
Metabolic pathways of berberine within the intestinal lumen.

Within this context, natural active constituents that reshape the gut microbiota are gaining significant attention. Berberine, the primary bioactive compound in *Coptis chinensis*, demonstrates exceptional promise due to its broad-spectrum antimicrobial, anti-inflammatory, and multi-target metabolic regulatory properties (Zhang et al., 2015). Notably, despite extremely low oral bioavailability (<1%) (Han et al., 2021; Chen et al., 2011), berberine is converted by gut microbiota into highly active metabolites (e.g., dihydroberberine) (Figure 1), substantially improving host metabolic phenotypes. This paradoxical efficacy stems from a core mechanism: berberine selectively enriches probiotics (e.g., butyrate producers) while suppressing pathogenic bacteria, thereby modulating microbiota-derived metabolites (e.g., SCFAs, bile acids). This regulation occurs through multi-organ axes (gut-liver, gut-brain) to influence host physiology (Habtemariam, 2020). Consequently, berberine serves as an exemplary model compound for studying natural product-microbiota interactions.

Bibliometric analysis is a systematic research method grounded in a quantitative paradigm, designed to provide a structured assessment of research trajectories, core themes, and dynamic evolution within a given scientific field. Leveraging mathematical and statistical tools, this approach delves into academic publications and their associated meta-data—including authorship patterns, institutional collaborations, keyword co-occurrence networks, and citation linkages—to map the intellectual landscape and developmental trends of the field. By doing so, researchers can systematically discern critical junctures and transformative pathways in the evolution of a discipline, track temporal shifts in research foci, and identify paradigm-shifting contributions and influential scholars (Ellegaard and Wallin, 2015). This analytical framework not only helps clarify established research trajectories and uncover theoretical gaps or methodological constraints but also offers a scientifically grounded orientation for future inquiry. More importantly, a bibliometric perspective enables the early identification of nascent intersections and underexplored directions, thereby providing data-driven support and a decision-making foundation for scientific planning, policy formulation, and resource allocation (Lazarides et al., 2025; Donthu et al., 2021). For academia and other stakeholders, this dual perspective—capable of delineating both the overarching trajectory of a discipline and its granular research details—fosters a more comprehensive grasp of the current state of the field and supplies robust support for evidence-informed scientific planning and cross-institutional collaboration (Akin et al., 2025).

Amidst the exponential growth of relevant literature, traditional review methods face limitations in systematically delineating the evolution of knowledge structures, interdisciplinary collaboration patterns, and emerging research frontiers within this field. Science mapping techniques, by quantifying relational networks among publications, provide a methodological breakthrough for elucidating the dynamic evolution of complex research landscapes (Chen, 2016). Among these tools, CiteSpace software leverages citation analysis and time-zone visualization to precisely identify evolutionary trajectories of research foci, pivotal literature hubs, and interdisciplinary characteristics (Chen et al., 2014; Forliano et al., 2021), demonstrating particular efficacy in detecting cutting-edge emergent trends (Burst Detection). In recent years, microorganisms have gradually become a research hotspot in the medical field, with the gut microbiota in particular being closely associated with the development of various diseases. Reshaping the gut microbial ecosystem can intervene in the progression of diverse diseases. For instance, berberine’s modulation of the gut microbiota can prevent and treat a range of conditions, including diabetes, obesity, inflammatory bowel disease, colorectal cancer, and even depression. Its mechanisms involve multiple microbiota – host co – metabolic pathways, such as those related to short – chain fatty acids (SCFAs), bile acids, tryptophan metabolites, and trimethylamine N – oxide (TMAO) (Cui H. et al., 2018; Zhang et al., 2024; Wang et al., 2018; Sun et al., 2022; Wang et al., 2021). However, the knowledge system in this field is highly fragmented. The same microbial genus or species often plays similar or different roles in different disease contexts. Research findings are scattered across journals in various disciplines, including pharmacology, microbiology, gastroenterology, and neuroscience. Traditional reviews, constrained by length and subjective viewpoints, find it challenging to systematically organize this interdisciplinary and multi – level knowledge network, let alone promptly capture rapidly emerging new research directions (Wang Y. et al., 2024).

In this study, we integrated data from both Chinese (CNKI) and international (WOSCC) databases and employed CiteSpace to conduct bibliometric and visualization analyses of articles focusing on the interaction between berberine and the gut microbiota, published between January 1, 2005 and January 31, 2025.

## 2 Data sources and methods of analysis

### 2.1 Materials and methods

#### 2.1.1 Search strategy

**Figure 2:**
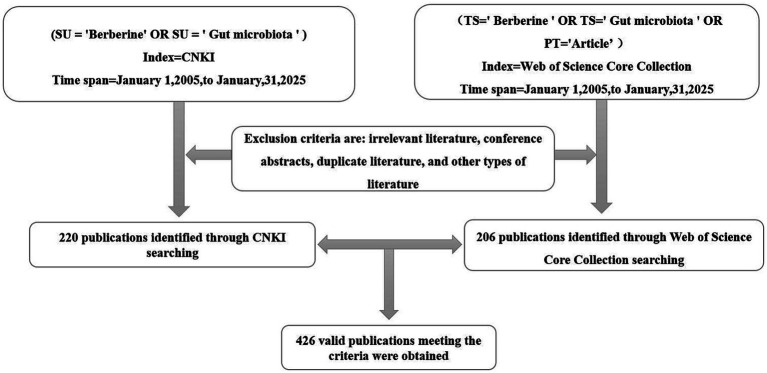
Search strategy of berberine on gut microbiota.

Systematic reviews are inherently qualitative and therefore vulnerable to interpretation bias. Bibliometric analysis, by contrast, relies on quantitative techniques, thereby mitigating this limitation. This approach enables scholars to examine large volumes of literature with greater transparency and reproducibility than conventional review methods (Wang C. et al., 2024). To this end, a systematic literature search was conducted utilizing the advanced search functions of both the CNKI and the WOSCC. Boolean operators (AND, OR) were employed to combine various keyword permutations pertaining to the interaction between berberine and the gut microbiota. The search timeframe was restricted to publications from January 1, 2005, to January 31, 2025, as illustrated in Figure 2, with the data collection executed on February 20, 2025.

For the CNKI database, a topic search was performed using the following strategy: (SU = ‘黄连素’ OR SU = ‘肠道菌群’ OR SU = ‘肠道微生物’) AND (SU = ‘小檗碱’ OR SU = ‘肠道菌群’ OR SU = ‘肠道微生物’). The language was limited to Chinese. Documents such as book chapters, conference proceedings, and notes were excluded, as these sources typically lack the depth, rigorous peer-review process, or original research focus required for a comprehensive bibliometric analysis. This process yielded 220 eligible articles. In parallel, the WOSCC was queried using a topic search: (TS = ‘Berberine’ OR TS = ‘Gut microbiota’ OR PT = ‘Article’). Applying the same exclusion criteria resulted in a corpus of 206 relevant articles. Following the screening process, the final dataset was consequently refined to a total of 426 papers. A comprehensive literature search was conducted across the CNKI and WOSCC databases to identify publications investigating the effects of berberine on gut microbiota (Figure 2). The search encompassed studies published between January 1, 2005, and January 31, 2025, with data collection finalized on February 20, 2025. Following removal of duplicate records and non-journal publications (e.g., conference abstracts), 220 articles from Chinese sources and 206 articles from English-language sources were retained for subsequent analysis.

#### 2.1.2 Analysis tools

For the bibliometric analysis, CiteSpace (version 6.4. R2 Advanced) was employed to systematically examine the literature published between January 1, 2005, and January 31, 2025. This software is widely recognized for its capacity to integrate diverse bibliographic data and apply multivariate, time-dependent, and visualization techniques, thereby enabling the automatic generation of knowledge maps that reveal research hotspots and evolving trends within a given scientific domain over a specified timeframe (Chen et al., 2014). The analysis was configured with the following parameters: a one-year time slice was used to capture temporal dynamics; the selection criterion was based on the g-index with a scaling factor of K = 25, which modulates the number of knowledge units extracted by adjusting the inclusion or exclusion of nodes. In each time slice, the top 50 most frequent items (Top N) and the top 10% of items (Top N%) were retained. To enhance network clarity—particularly given the large volume of publications—the pruning process was optimized using the Pathfinder algorithm combined with “Pruning the merged network.

The resulting analyses encompassed several dimensions: annual publication trends, collaborative networks among countries and institutions, co-authorship patterns, keyword co-occurrence with clustering, burst keyword detection, and co-citation reference analysis. These findings were visualized as knowledge maps, in which key nodes and their interconnecting links can be interpreted and examined. Within these maps, each node represents an entity under investigation—such as a country, institution, author, or keyword—with its size reflecting the frequency of occurrence: the larger the node, the higher the frequency. Furthermore, the color and thickness of the rings within a node convey the temporal distribution of occurrences across different periods. In the generated network visualizations, nodes outlined in purple denote high betweenness centrality (BC). A centrality value≥0.1 is conventionally regarded as indicative of node significance. Betweenness centrality, a fundamental graph-theoretic property, quantifies a node’s importance in mediating information flow within networks. Cluster map evaluation employs two key metrics: modularity (Q) and mean silhouette coefficient (S). A Q-value>0.3 typically signifies substantial modular community structure, with higher values reflecting denser network topology and more effective clustering outcomes. Similarly, the clustering is considered reasonable if S surpasses 0.5, whereas a value exceeding 0.7 denotes highly reliable clustering results. The transformed bibliometric data underwent comprehensive visualization and analysis encompassing: publication volume, author co-occurrence, institutional co-occurrence, keyword co-occurrence, keyword clustering, keyword burst detection, and co-citation analysis. Keyword burst detection is particularly valuable for identifying abrupt surges of research interest in specialized domains (Chen, 2006; Nelson et al., 2017).

## 3 Results

### 3.1 Analysis of papers by publication year

**Figure 3:**
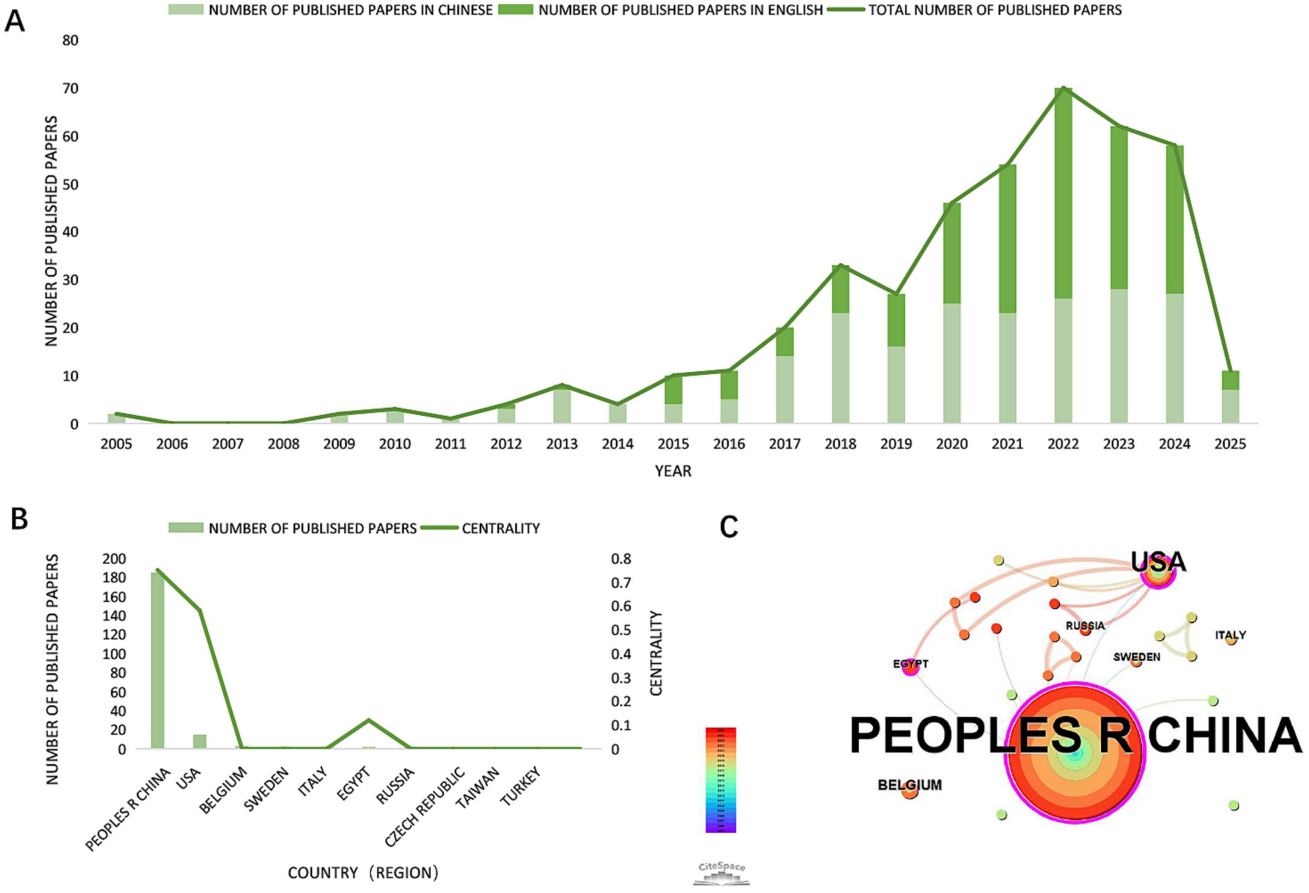
Number of publications and country/regional distribution. **(A)** Visual analysis of papers in berberine-gut microbiota research by publication year; **(B)** top 10 countries/regions with the highest publication output in berberine-gut microbiota research; **(C)** map of countries in berberine-gut microbiota research.

Analyzing publication trends in relevant literature provides critical insights into the current research landscape, growth trajectory, and future directions of a field. Visualization of annual publication volume for berberine-gut microbiota interaction research (Figure 3A) reveals an exponential growth” from 2019 to 2022. This acceleration is closely linked to breakthrough advances in understanding the mechanistic roles of gut microbiota-derived metabolites (SCFAs/TMAO) (Tremaroli and Bäckhed, 2012; Sun et al., 2017). Notably, a deceleration in publication growth emerged after 2023, likely reflecting the field’s need for cross-disciplinary technological integration (e.g., multi-omics analyses) to address increasingly complex mechanistic questions. We note that literature data for 2025 were incomplete as of January 31, 2025, however, this limitation does not compromise the validity of the observed trends. Collectively, annual publication output demonstrates sustained growth in berberine-gut microbiota interaction research.

### 3.2 Analysis of cooperation among countries/regions

China accounts for 82.20% of global research output on berberine-gut microbiota interactions, representing the dominant contributor, followed by the United States and Belgium (Figure 3B). The international collaboration network consists of 24 nodes and 24 links, exhibiting a density of 0.087 (Figure 3C). China and the United States, highlighted in purple, occupy central positions within the network, indicating their extensive collaborative ties with other nations. Although China leads in publication volume, both China and the United States demonstrate the highest betweenness centrality (BC ≥ 0.58), signifying their critical roles in maintaining network connectivity. Notably, Egypt, while ranking third in publication volume, exhibits a betweenness centrality of 0.12, suggesting its emergence as a potential hub for international collaboration (Figure 3C).

### 3.3 Analysis of institutions

Within the CNKI database, Beijing University of Chinese Medicine (BUCM) produced the highest number of publications (*n* = 15). In the WOSCC, Chinese Academy of Medical Sciences-Peking Union Medical College (PUMC) contributed the most publications (*n* = 29). Other high-output institutions were exclusively academic or research entities, including the China Academy of Chinese Medical Sciences and Shanghai Jiao Tong University (Table 1), reflecting concentrated scholarly activity in these settings.

**Table 1:** Top 10 research institutions in terms of publication output in CNKI and WOSCC.

No.	CNKI		WOSCC	
	Institution	Count	Institution	Count
1	Beijing University of Chinese Medicine	15	Chinese Academy of Medical Sciences-Peking Union Medical College	29
2	China Academy of Chinese Medical Sciences	7	Chinese Academy of Sciences	14
3	Chinese Academy of Medical Sciences	6	Shanghai Jiao Tong University	13
4	Chengdu University of Traditional Chinese Medicine	6	Institute of Materia Medica-CAMS	10
5	Xiamen University	6	China Academy of Chinese Medical Sciences	10
6	Southwest University	5	Shanghai University of Traditional Chinese Medicine	9
7	Nanjing University of Chinese Medicine	5	Beijing University of Chinese Medicine	9
8	Shanghai Jiao Tong University	5	Guangzhou University of Chinese Medicine	9
9	Guangdong Pharmaceutical University	5	Chengdu University of Traditional Chinese Medicine	8
10	Capital Medical University	4	Chinese University of Hong Kong	7

**Figure 4:**
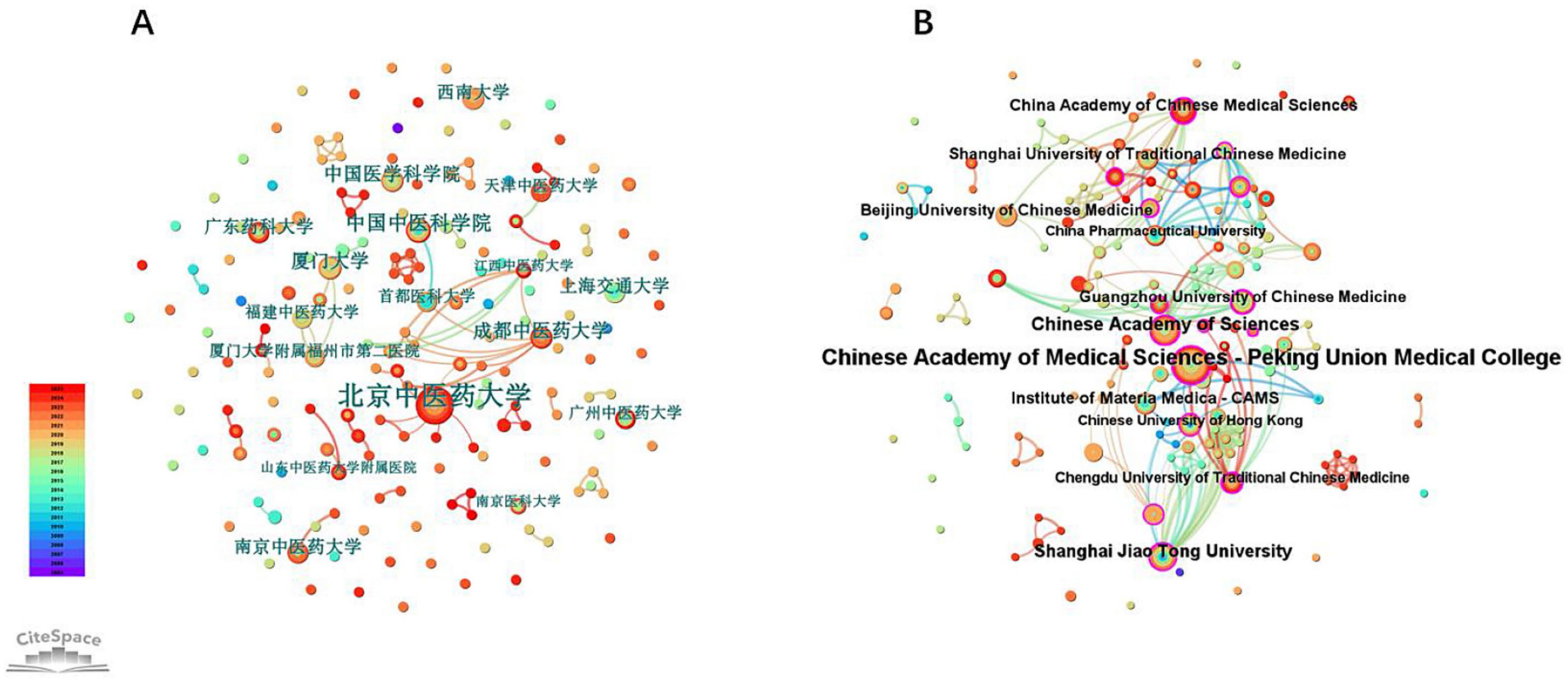
Analysis of institutional collaboration network in berberine-gut microbiota interaction research. **(A)** Map of institutions (Data source: CNKI); **(B)** map of institutions (Data source: WOSCC).

The CNKI co-authorship network (Figure 4A) exhibits sparse connectivity (Density = 0.0056) with 173 nodes and 84 edges, where Beijing University of Chinese Medicine (BUCM) emerges as a key research hub. Although BUCM demonstrates prominence, inter-institutional collaborations remain fragmented. In contrast, the WOSCC network (Figure 4B) demonstrates significantly higher structural integration (Density = 0.023) with 148 nodes and 250 edges, reflecting greater global research synergy. However, collaborative activities remain predominantly concentrated within Chinese institutions, including major biomedical centers like the Chinese Academy of Medical Sciences (CAMS) and Shanghai Jiao Tong University, with no international nodes achieving central network positions.

Although Peking Union Medical College (PUMC) and Beijing University of Chinese Medicine (BUCM) function as dual core institutions (Table 1), their inter-institutional collaboration frequency remains suboptimal, suggesting untapped synergistic potential. Critically, the WOSCC network reveals no participation from European or North American institutions (Figure 4B), contrasting with China’s substantial contribution to global output (82.20% of publications). Collaborative linkages occur primarily among institutions focused on traditional Chinese medicine (TCM) and biomedical research, with minimal involvement from Western academic or industrial sectors.

### 3.4 Analysis of authors and cocited authors

Table 2 profiles the top 10 most productive authors across Chinese and English databases. In CNKI, Wu Qiqi and Zhou Qiang co-led the ranking with 4 publications each, while the WOSCC was dominated by Wang Yan (9 publications) and Jiang Jian-Dong (8 publications). Notably, academician Jiang Jian-Dong’s research group demonstrated sustained high productivity across both Chinese and international literature platforms. Their foundational work on metabolic regulation via SCFAs and gut microbiota-derived L-DOPA for Parkinson’s disease therapy constitutes cornerstone contributions to the field (Wang et al., 2021; Ma et al., 2022).

**Table 2:** The top 10 authors of berberine-gut microbiota research in CNKI and WOSCC.

No.	CNKI		WOSCC	
	Author	Count	Author	Count
1	WU, Qiqi	4	Wang, Yan	9
2	Zhou, Qiang	4	Jiang, Jian-Dong	8
3	Yang, Qing	3	Fu, Jie	6
4	Weng, Xiaogang	3	Pan, Li-Bin	4
5	Men, Wei	3	Wu, Chongming	4
6	Gong, Zipeng	3	Ma, Shu-Rong	4
7	He, Yasha	3	Chen, Haitao	4
8	Zhu, Xiaoxing	3	Yang, Yanan	3
9	Chen, Ying	3	He, Chi-Yu	3
10	Jiang, Jian-Dong	3	Feng, Ru	3

**Figure 5:**
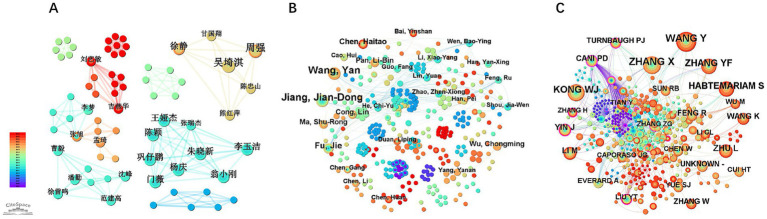
Analysis of authors and collaborative networks in berberine-gut microbiota research. **(A)** Map of authors (Data source: CNKI); **(B)** map of authors (Data source: WOSCC); **(C)** map of co-cited authors in berberine-gut microbiota research (Data source: WOSCC).

The CNKI network (Figure 5A) has 295 nodes (authors) and 392 links (collaborations), with a density of 0.009. The WOSCC network (Figure 5B) has 355 nodes and 776 links, and a slightly higher density of 0.012. But it’s still much lower than that of a healthy collaboration network (usually > 0.1). No nodes with a betweenness centrality≥0.1 (maximum BC = 0.07) or purple-bordered nodes emerged in the two networks, indicating that no core collaborative clusters with a pivotal role have formed in this field.

This contrasts with the cited-author network (Figure 5C), where international scholars like CANI PD and TURNBAUGH PJ serve as hubs for cross-team collaboration due to their high centrality (BC ≥ 0.1). Notably, the research team of Jiang Jian-Dong, despite its seminal contributions to mechanistic studies (Table 3), has not achieved hub-level influence in collaborative networks. This further confirms the previously mentioned issue of untapped inter-institutional collaboration potential.

**Table 3:** The top 10 cited references of berberine-gut microbiota research.

No.	Count	Cited reference	Centrality	Cited reference
1	34	Habtemariam S, 2020, PHARMACOL RES, V155, P0, DOI: 10.1016/j.phrs.2020.104722	0.38	Tremaroli V, 2012, NATURE, V489, P242, DOI: 10.1038/nature11552
2	24	Zhang YF, 2020, NAT COMMUN, V11, P0, DOI: 10.1038/s41467-020-18414-8	0.31	Yue SJ, 2019, AM J PHYSIOL-ENDOC M, V316, PE73, DOI: 10.1152/ajpendo.00256.2018
3	23	Wang Y, 2017, METABOLISM, V70, P72, DOI: 10.1016/j.metabol.2017.02.003	0.25	Cani PD, 2008, DIABETES, V57, P1470, DOI: 10.2337/db07-1403
4	21	Zhang W, 2019, BIOMED PHARMACOTHER, V118, P0, DOI: 10.1016/j.biopha.2019.109131	0.22	Lan JR, 2015, J ETHNOPHARMACOL, V161, P69, DOI: 10.1016/j.jep.2014.09.049
5	18	Zhu L, 2018, ATHEROSCLEROSIS, V268, P117, DOI: 10.1016/j.atherosclerosis.2017.11.023	0.22	Chang WG, 2015, BIOCHEM CELL BIOL, V93, P479, DOI: 10.1139/bcb-2014-0107
6	17	Zhang X, 2015, SCI REP-UK, V5, P0, DOI: 10.1038/srep14405	0.19	Zhang X, 2015, SCI REP-UK, V5, P0, DOI: 10.1038/srep14405
7	16	Cui HT, 2018, FRONT PHARMACOL, V9, P0, DOI: 10.3389/fphar.2018.00571	0.17	Zhang W, 2019, BIOMED PHARMACOTHER, V118, P0, DOI: 10.1016/j.biopha.2019.109131
8	15	Wu M, 2020, FRONT PHARMACOL, V11, P0, DOI: 10.3389/fphar.2020.00223	0.16	Dehau T, 2023, MSYSTEMS, V8, P0, DOI: 10.1128/msystems.01239-22
9	15	Wang Y, 2017, THERANOSTICS, V7, P2443, DOI: 10.7150/thno.18290	0.15	Derosa G, 2012, EXPERT OPIN BIOL TH, V12, P1113, DOI: 10.1517/14712598.2012.704014
10	13	Tian Y, 2019, DRUG METAB DISPOS, V47, P86, DOI: 10.1124/dmd.118.083691	0.14	Xie WD, 2011, PLOS ONE, V6, P0, DOI: 10.1371/journal.pone.0024520

### 3.5 Analysis of keywords and keyword clusters

Keywords represent highly condensed descriptors of core research themes. Table 4 integrates the top 10 high-frequency keywords from Chinese and English databases. Analysis reveals distinct research priorities: Chinese-language publications emphasize disease applications (e.g., diabetes hyperlipidemia therapeutic efficacy) whereas English-language literature focuses on mechanistic investigations (e.g., metabolism mechanism). Inflammation is as a shared research focus across both linguistic domains. Supporting evidence demonstrates that berberine: (1) effectively reduces mRNA expression of inflammatory cytokines while upregulating tight junction protein expression in Caco-2 cells thereby attenuating inflammatory responses (Ding et al., 2023); (2) alleviates dextran sulfate sodium (DSS)-induced ulcerative colitis in mice by restoring gut microbiota composition elevating unconjugated/secondary bile acids and activating FXR/TGR5 signaling pathways (Sun et al., 2023); and (3) significantly ameliorates colitis symptoms and colonic damage through IL-6 modulation and gut microbiota remodeling (Zhao et al., 2021).

**Table 4:** Top 10 keywords of berberine-gut microbiota research in CNKI and WOSCC.

No.	CNKI		WOSCC	
	Keyword	Count	Keyword	Count
1	Berberine	159	Gut microbiota	127
2	Gut microbiota	132	Inflammation	28
3	Diabetes	16	Metabolism	26
4	Mechanism of action	10	Obesity	25
5	Coptis	8	Ulcerative colitis	22
6	Hyperlipidemia	7	Mechanism	20
7	Inflammation	6	Microbiota	19
8	Therapeutic effect	6	Activation	17
9	Metformin	6	Insulin resistance	15
10	Inflammatory factor	6	Disease	14

**Figure 6:**
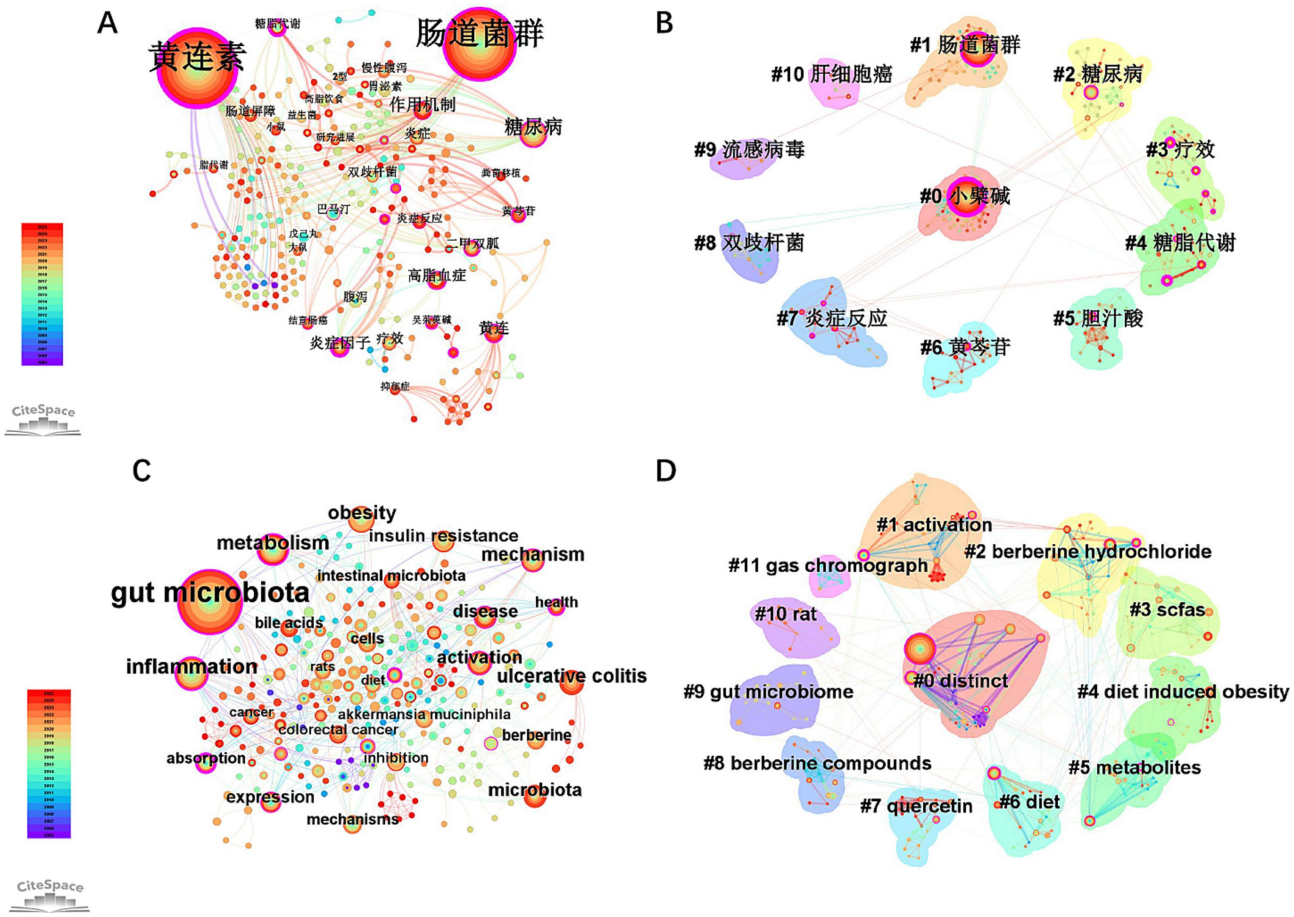
Analysis of keywords and keyword clusters networks in berberine-gut microbiota research. **(A)** Map of coreferenced keywords (Data source: CNKI); **(B)** map of the coreferenced keyword clusters (Data source: CNKI); **(C)** map of coreferenced keywords (Data source: WOSCC); **(D)** map of the coreferenced keyword clusters (Data source: WOSCC).

The CNKI keyword network consists of 260 nodes connected by 468 links (Figure 6A). With a relatively low density (Density = 0.0139), the core of the network revolves around keywords such as berberine (黄连素), gut microbiota (肠道菌群), diabetes (糖尿病) and hyperlipidemia (高脂血症) highlighting the high concentration on clinical efficacy research. The emerging keyword “fecal microbiota transplantation” (emerging from 2021 to 2025) has yet to form a tightly-knit cluster, indicating a stage of exploration. The resulting clustering map reveals 12 distinct clusters: #0 (berberine) (黄连素), #1 (gut microbiota) (肠道菌群), #2 (diabetes) (糖尿病), #3 (carbohydrate and lipid metabolism) (糖脂代谢), #4 (therapeutic effect) (疗效), #5 (bile acid) (胆汁酸), #6 (baicalin) (黄芩苷), #7 (inflammatory response) (炎症反应), #8 (Bifidobacterium) (双歧杆菌), #9 (influenza virus) (流感病毒), #10 (hepatocellular carcinoma) (肝细胞癌). The analysis of these clusters demonstrates an S-value of 0.9692 and a Q-value of 0.8262. All clusters have an S-value greater than 0.7 and a Q-value exceeding 0.3, confirming the rationality of the clustering. These 12 keyword clusters accurately reflect the distribution of research themes in the analyzed literature (Figure 6B).

For WOSCC, the keyword network is composed of 277 nodes connected by 715 links (Figure 6C). The core of the network is centered around keywords such as gut microbiota, inflammation and metabolism. The presence of purple-bordered nodes and the strong interconnections among nodes are notable. With a higher network complexity (Density = 0.0187), keywords such as gut microbiota, inflammation and metabolism exhibit high centrality. This underscores their pivotal roles in metabolism and inflammation mechanisms. The resulting clustering map reveals the following 12 distinct clusters: #0 (distinct), #1 (activation), #2 (berberine hydrochloride), #3 (scfas), among others (Figure 6D). The analysis of these clusters in the map shows an S-value of 0.8913 and a Q-value of 0.7178. All clusters have an S-value greater than 0.7 and a Q-value exceeding 0.3, confirming the rationality of the clustering. Therefore, these 12 keyword clusters also accurately reflect the distribution of research themes in the analyzed literature.

### 3.6 Keywords with the most robust citation bursts

**Figure 7:**
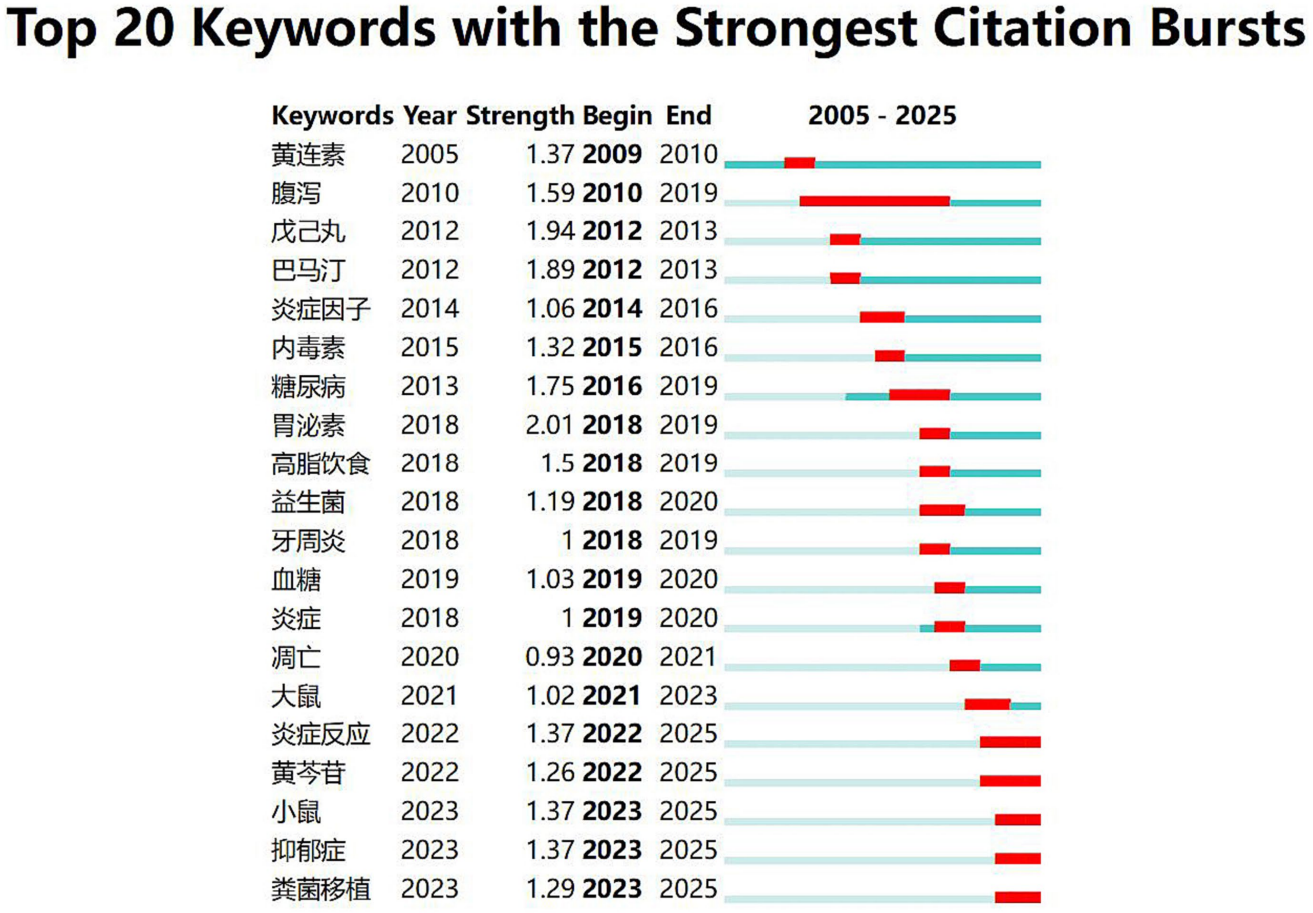
Top 20 keywords with the strongest citation bursts in berberine-gut microbiota research (data source: CNKI).

Analysis of 20 burst terms in the CNKI database (Figure 7) reveals three evolutionary phases: From 2010–2019, research focused predominantly on diarrhea (腹泻), reflecting traditional berberine applications for gastrointestinal disorders. During 2014–2020, emerging terms including inflammatory factors (炎症因子), diabetes (糖尿病), and probiotics (益生菌) signaled strengthened mechanistic linkages to metabolic diseases. The 2021–2025 period features emerging bursts for depression (抑郁症), FMT (粪菌移植), baicalin, and rat/mouse (experimental models). Notably, *Coptis chinensis* (huanglian), a primary component in anti-diabetic Chinese patent medicines like Jinqi Jiangtang Tablets, Tangmaikang Capsules, and Xiaoke Ping Tablets, is frequently combined with *Astragalus membranaceus* (huangqi). This herb pair modulates gut microbiota composition (particularly *Bacteroide*s and *Parabacteroides* abundance), thereby improving hyperlipidemia (Gao, 2024). With baicalin emerging as a key term, berberine-baicalin co-administration demonstrates therapeutic potential for applications beyond metabolic disorders. The prominence of depression (抑郁症) underscores the gut-brain axis as a research priority, while FMT highlights advances in precision microbiota interventions. Collectively, CNKI burst terms trace a progression from symptom-focused (diarrhea) to mechanism-driven (inflammation/metabolism) to interdisciplinary domains (neuro-microbiota interactions, FMT-integrated therapies).

**Figure 8:**
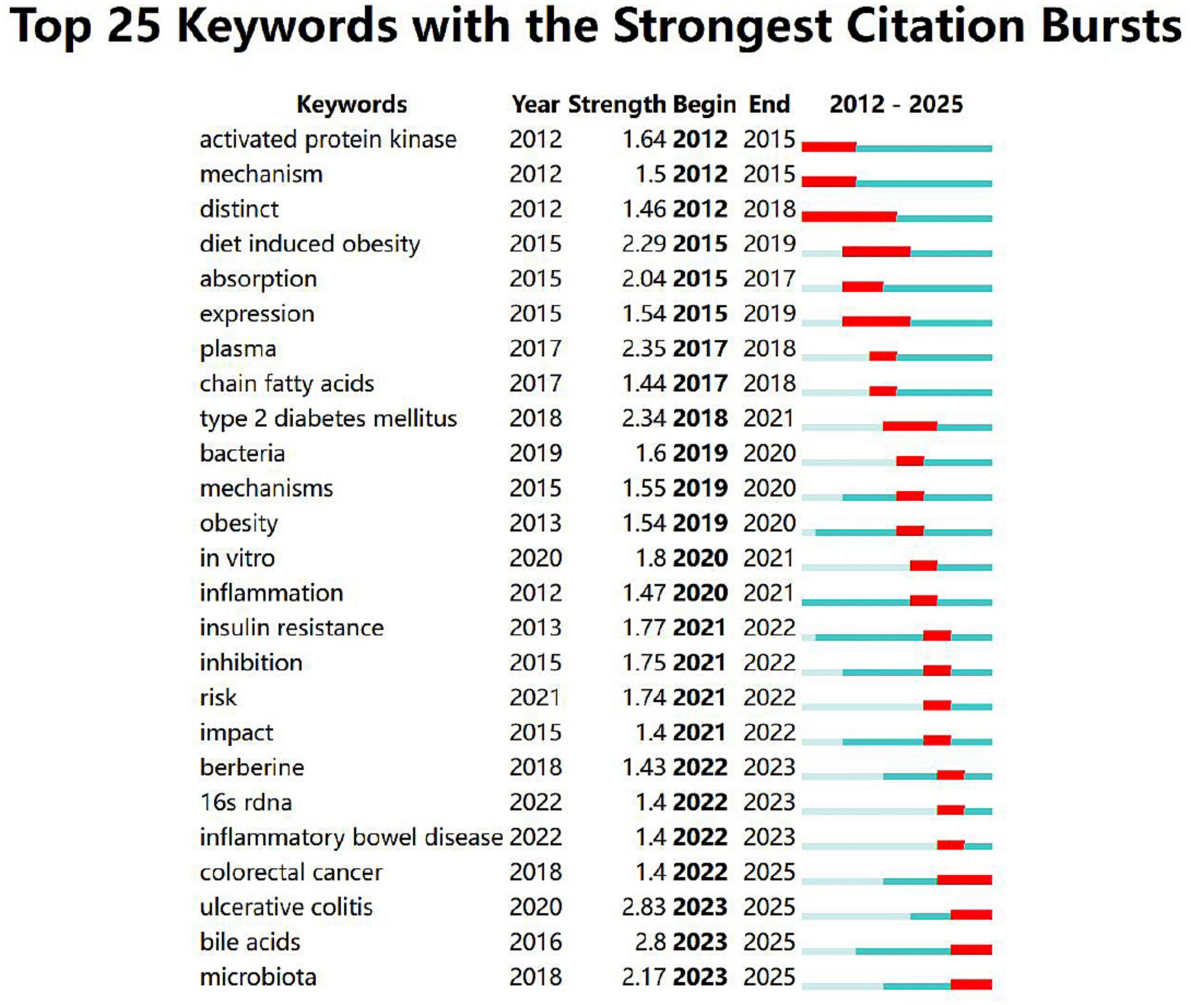
Top 25 keywords with the strongest citation bursts in berberine–gut microbiota research (Data source: WOSCC).

In WOSCC, analysis of 25 burst terms (Figure 8) identifies disease mechanism refinement as the dominant trend. Sustained bursts for ulcerative colitis, colorectal cancer, and bile acids indicate intensified investigation of microbiome-immune-metabolism crosstalk. These terms collectively reflect a shift from singular disease associations toward multi-scale regulatory mechanisms in microbiota-host interactions. Specifically, bile acids and activation denote molecular/cellular pathway exploration, while persistent co-emergence of colorectal cancer and ulcerative colitis reveals growing interest in inflammation-cancer transitions. The burst status of microbiota further suggests microbiome-targeted precision interventions represent emerging research priorities. Consequently, FMT and precision microbiome modulation constitute key future directions for berberine-based therapeutics.

### 3.7 Co-citation network of references

**Figure 9:**
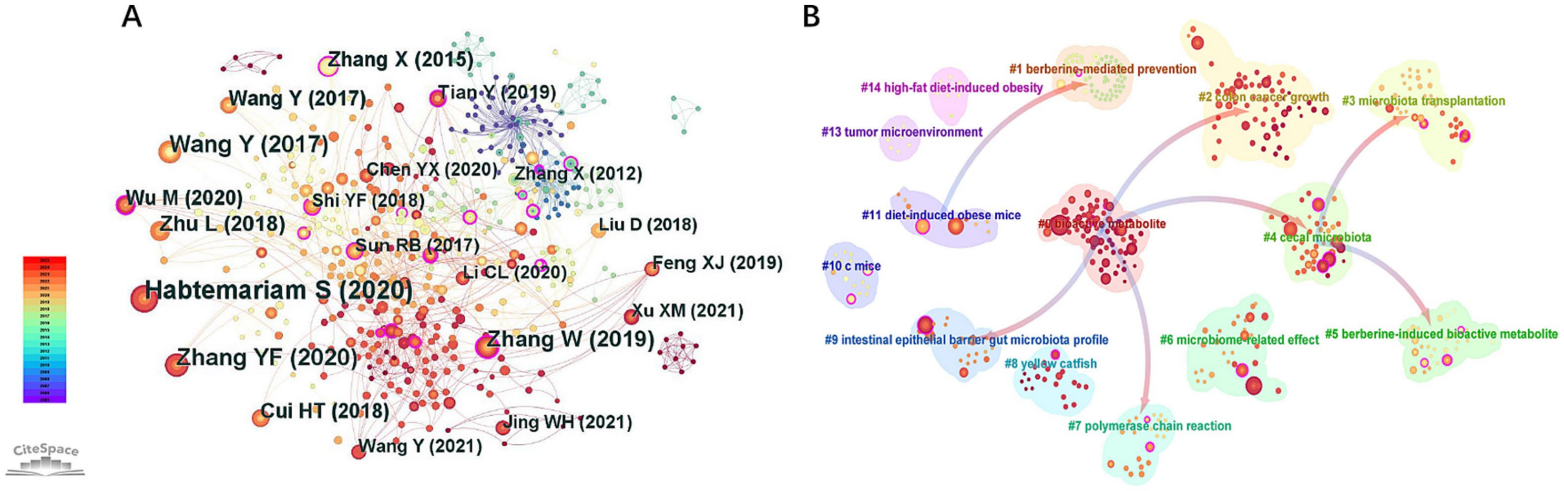
References networks in berberine-gut microbiota research. **(A)** Map of cited references; **(B)** cluster dependency map of the reference.

The co-citation network reveals the structure and evolution of knowledge foundation in the field by clustering highly interrelated literature groups. Table 3 lists the top 10 references ranked by citation frequency and BC, while Figure 9 visualizes the co-citation network and citation paths between clusters within the WOSCC database.

Analysis of highly influential publications in Table 3 reveals two categories forming the core knowledge base. Habtemariam (2020), the most cited work (Top 1), systematically elucidates the molecular mechanisms through which berberine modulates the microbiota-metabolism-immune network via gut microbiota remodeling. This work emphasizes the multi-target therapeutic potential of berberine for metabolic/inflammatory diseases while highlighting that inter-individual microbiota variations may contribute to efficacy heterogeneity. Tremaroli (2012), exhibiting the highest betweenness centrality (BC = 0.38), establishes gut microbiota as a metabolic organ influencing host metabolism through energy regulation, immune activation, and signaling molecules, thereby laying the theoretical groundwork for microbiota-targeted therapies against obesity and diabetes. Zhang et al. (2015), ranked Top 6 in both citation frequency and betweenness centrality, experimentally validates in animal models that berberine ameliorates metabolic endotoxemia through microbiota modulation. Publications with high BC values (≥0.15) predominantly focus on microbiota-host metabolic interactions, such as SCFAs, bile acids, and TMAO pathways, reinforcing the theoretical basis for core research foci like metabolism and inflammation identified through keyword analysis (Table 4; Figures 6A,B).

The co-citation network generated 14 significant clusters (Q = 0.8121; S = 0.9127). Pathfinder-based simplification (retaining top 50% inter-cluster citation pathways; Figure 9B) highlights key knowledge dependencies: Cluster #0 (bioactive metabolite) cites literature from #2 (colon cancer growth-CRC mechanisms), #4 (cecal microbiota-compositional analysis), #7 (polymerase chain reaction-molecular techniques), and #9 (intestinal epithelial barrier-gut barrier function), indicating these clusters form the foundation for metabolite research. This dependency structure reveals the logical progression from fundamental mechanisms (microbiota composition, analytical techniques) → functional metabolite studies (SCFAs/bile acids) → disease applications (CRC, metabolic disorders), aligning with the “colorectal cancer→bile acids→microbiota” frontier trend identified through burst detection (Figure 8). Future studies should prioritize integrating Cluster #7 (molecular techniques) with Cluster #0 (metabolite applications) to advance multi-omics approaches for targeted modulation of microbiota-derived metabolites.

### 3.8 Journal category analysis

**Figure 10:**
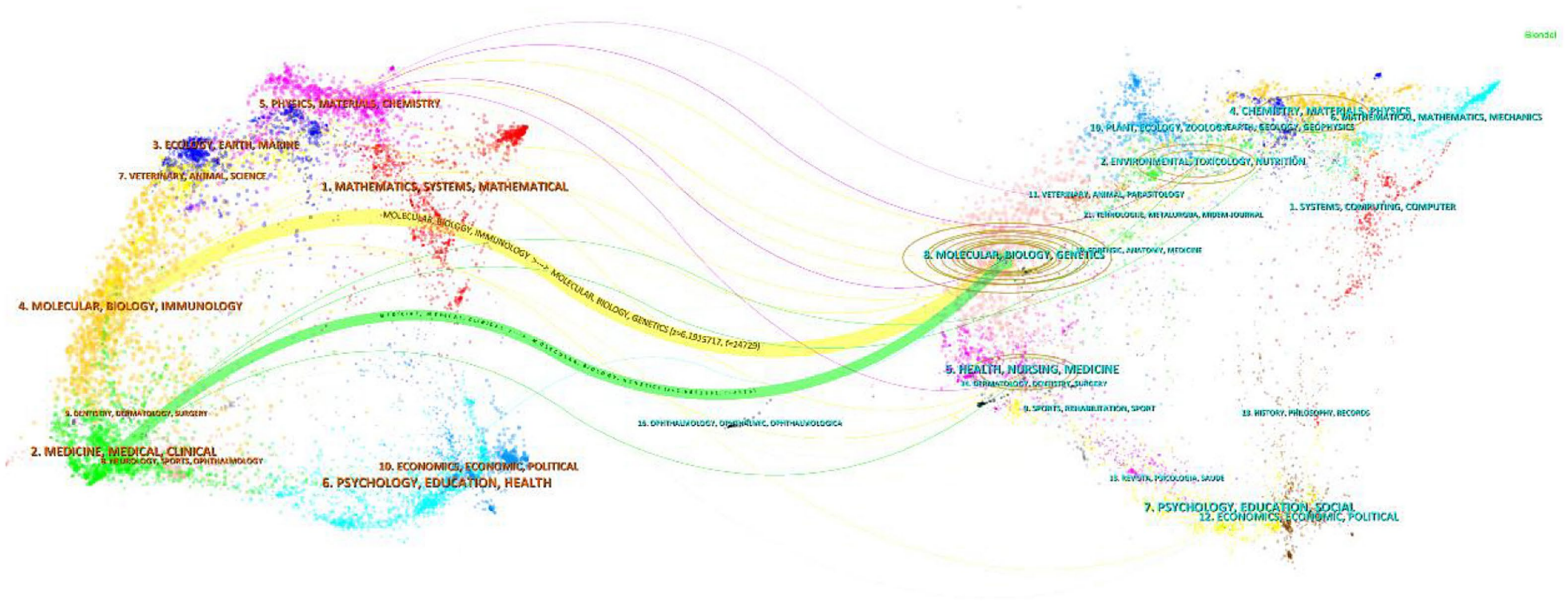
Overlay map of journals in berberine-gut microbiota research.

The journal overlay map (Figure 10) visualizes the knowledge flow between fields through citation curves. On the left are the citing journals, and on the right are the cited journals. The length of the horizontal axis of an ellipse indicates the number of active authors in the field, while the vertical axis represents the average annual number of articles published by the journal. The area of the ellipse is proportional to the annual number of publications, and the width of the curves reflects the strength of citation frequency.

The citing journals are mainly in the fields of medicine, molecular biology, immunology, physics, materials science, and chemistry. Correspondingly, the cited literature primarily comes from journals in molecular biology, biology, genetics, anatomy, environmental science, toxicology, nutrition, and chemistry. The citation curves from basic disciplines (molecular biology, genetics) to applied disciplines (medicine, pharmacy) are the densest, confirming that research on “microbiota-metabolism” mechanisms drives clinical translation.

## 4 Discussion

### 4.1 Analysis of the current state and evolving research trends

This study presents the first systematic bibliometric analysis of research on the berberine–gut microbiota interaction from 2005 to 2025 using CiteSpace, delineating the macro-level knowledge structure and evolutionary trajectory of the field. Our analysis reveals a marked upward trend in publication output (Figure 3A), with a notable surge after 2019, indicating a transition from initial phenomenological observations toward in-depth mechanistic investigation and clinical translation. China dominates the research landscape, contributing 82.20% of the total publications (Figure 3B), underscoring its substantial investment and research prowess rooted in traditional medicine theory. Core research institutions are predominantly leading universities and academies of Chinese medicine and medical sciences (Table 1). However, the low density of institutional and collaborative networks (CNKI: 0.0056; WOSCC: 0.023) suggests that potential for interdisciplinary and cross-regional collaboration remains under exploited.

Keywords and co-citation analyses not only confirm long-standing core research themes such as “gut microbiota” “diabetes” “inflammation” and “metabolism” but more importantly burst detection identifies emerging shifts in research fronts (Figures 7, 8). The emergence of terms such as “depression” “fecal microbiota transplantation (FMT)” and “baicalin” in Chinese literature alongside the rising prominence of “colorectal cancer (CRC)” “ulcerative colitis (UC)” “bile acids” and “microbiota” in English publications collectively sketches a future roadmap for the field. This evolution reflects a movement from a focus on metabolic diseases alone toward more complex systemic disorders such as neuropsychiatric and gastrointestinal cancers; from describing microbial community structures toward elucidating the functional roles of microbiota-derived metabolites (e.g., bile acids); and from single-drug interventions toward precision microecological strategies like FMT-based combination therapies.

The co-citation network (Figure 9; Table 3) further unveils two foundational knowledge pillars underpinning these research hotspots: (1) the central role of microbial metabolites (e.g., Habtemariam S, 2020; Tremaroli V, 2012), establishing SCFAs, bile acids, and TMAO as key mediators of berberine’s effects; and (2) cross-organ immune and metabolic interactions (e.g., Zhang X, 2015), providing a theoretical basis for holistic physiological concepts such as the gut–liver and gut–brain axes. The journal overlay map (Figure 10) visually underscores the highly interdisciplinary nature of the field, showing vigorous knowledge exchange between basic disciplines (e.g., molecular biology, chemistry) and applied sciences (e.g., medicine, pharmacology), characteristic of a cutting-edge translational research domain.

Nevertheless, our analysis also highlights a critical paradox: despite China’s dominant productivity, it has not yet cultivated core authorial teams with international hub influence (Figure 5). This finding underscores the need for enhanced global collaboration, deeper mechanistic exploration, and more robust clinical validation in future research endeavors.

### 4.2 Linking gut microbial ecology to host phenotypes: how berberine exerts its therapeutic effects through microbiota modulation

Keywords co-occurrence analysis identifies “metabolism” “inflammation” and “bile acids” as enduring high-frequency terms (Table 4) while burst detection highlights “ulcerative colitis” “colorectal cancer” “depression” and “bile acids” as emerging hotspots in recent years (Figures 7, 8). Moreover the document co-citation network links these hotspots to foundational studies (Table 3). Here we integrate these core and frontier directions to dissect the molecular mechanisms by which berberine prevents and treats diseases through gut microbiota modulation aiming to consolidate current knowledge and inform future research.

#### 4.2.1 Berberine reshapes gut microbial composition and preserves intestinal barrier integrity

The keyword co-occurrence network (Figure 6) reveals a strong link between “gut microbiota” and “inflammation” a connection supported by highly central co-cited works such as Tremaroli (2012) and Zhang X (2015), which established the conceptual framework for the microbiota–barrier–inflammation axis. Berberine selectively enriches SCFAs-producing beneficial bacteria, including *Akkermansia muciniphila*, *Faecalibacterium prausnitzii*, *Bifidobacterium*, and *Lactobacillus* spp., while suppressing opportunistic pathogens such as *Escherichia/Shigella* and *Enterobacter* spp. (Karaosmanoglu et al., 2014; Lv et al., 2015; Chen et al., 2015; Cernáková and Kostálová, 2002; Yue et al., 2019). This compositional shift is functionally important. *A. muciniphila*, for instance, has been shown to reinforce the intestinal barrier by increasing mucus thickness and enhancing tight junction proteins including occludin and ZO-1 (Habtemariam, 2020). By promoting the expansion of these barrier-protective microbes, berberine limits the translocation of LPS and other endotoxins into circulation, thereby attenuating metabolic endotoxemia and systemic inflammation—a mechanism that underpins its therapeutic effects across both metabolic and inflammatory diseases (Zhang et al., 2015; Cui H. et al., 2018; Chen et al., 2015; Cui H. X. et al., 2018).

#### 4.2.2 Modulation of metabolic diseases (obesity, hyperlipidemia, diabetes mellitus): targeting microbial metabolic pathways

“Metabolism” ranks among the most frequent keywords in the WOSCC analysis (Table 4), and “bile acids” has emerged as a sustained burst term in recent years (Figure 8). Co-cited references, particularly Tremaroli (2012) and Habtemariam (2020), provide a systematic framework for understanding how microbial metabolites influence host metabolism. Consistent with this, the metabolic benefits of berberine can be attributed to its precise regulation of specific microbial pathways. First, through the SCFA pathway, berberine markedly increases the abundance of butyrate-producing bacteria such as *Blautia* and *Butyricicoccus* (Zhang et al., 2019; Zhu et al., 2018; Shi et al., 2018; Wang et al., 2018; Sun et al., 2018). Once absorbed, SCFAs like butyrate activate G-protein-coupled receptors (GPR41/GPR43) on intestinal L cells to stimulate GLP-1 secretion, thereby improving insulin sensitivity and glucose homeostasis (Habtemariam, 2020; Sun et al., 2017; Yadav et al., 2013; Shan et al., 2013). In parallel, butyrate functions as a histone deacetylase (HDAC) inhibitor to directly modulate host gene expression and suppress inflammation (Shan et al., 2013). Second, berberine alters the bile acid metabolic activity of the gut microbiota, leading to an increased proportion of secondary bile acids such as ursodeoxycholic acid (UDCA) (Sun et al., 2017). This reshaped bile acid pool activates the intestinal farnesoid X receptor (FXR) and G-protein-coupled bile acid receptor 1 (TGR5), which in turn regulate genes involved in glucose and lipid metabolism, contributing to hepatic protection and metabolic improvement (Sun et al., 2017; Wang et al., 2018). The convergence of this mechanism with the “bile acids” burst term highlights its emerging role as a central node in berberine’s metabolic actions. Third, berberine and its gut microbial metabolite dihydroberberine inhibit the activity of bacterial choline TMA-lyase (CutC/D), a key enzyme in TMA production. This interference reduces the conversion of choline to TMA and subsequently lowers circulating levels of its liver-derived oxidation product, TMAO—an independent risk factor for atherosclerosis (Shi et al., 2018). These multifaceted actions underscore the role of berberine in modulating metabolic diseases such as obesity, hyperlipidemia, and diabetes through targeted regulation of microbial metabolic pathways (Zhang et al., 2015; Habtemariam, 2020; Shan et al., 2013; Liu et al., 2018). Notably, the efficacy of BBR in ameliorating metabolic syndrome matches metformin, with evidence suggesting potential synergy (Zhang et al., 2019; Poroyko et al., 2016; Bech-Nielsen et al., 2012; Sepp et al., 2014; Larsen et al., 2010; Pedersen et al., 2016; Wu et al., 2010). Berberine suppresses lipogenesis and enhances fatty acid oxidation through both AMPK-dependent and AMPK-independent mechanisms, thereby reducing liver weight, blood lipid, and cholesterol levels in obese animal models (Yang et al., 2019).

#### 4.2.3 Targeting inflammatory disease: ulcerative colitis

Burst detection analysis reveals that “ulcerative colitis” (UC) persists as a prominent keyword with strong citation bursts in the WOSCC through 2025 (Figure 8). With a burst strength of 2.83 during 2023–2025—the highest among the top 20 burst keywords—this trend underscores the emerging significance of UC-related research in the context of berberine and gut microbiota. This frontier status is further corroborated by the high citation frequency of Cui HT (2018) in the co-citation network (Table 3). In UC, the therapeutic effects of berberine extend well beyond its conventional antimicrobial and barrier-protective functions. Evidence indicates that berberine modulates intestinal immune homeostasis by increasing the proportion of regulatory T cells (Tregs) while suppressing excessive activation of pro-inflammatory T helper 17 cells (Th17), thereby restoring immune tolerance in the colonic mucosa (Cui H. et al., 2018). This immunomodulatory effect operates in concert with microbiota remodeling: the enrichment of SCFAs, particularly butyrate, serves as a key signaling mechanism that promotes Treg differentiation and limits local inflammation. In parallel, berberine downregulates classical inflammatory pathways such as NF-κB, directly reducing the production of pro-inflammatory cytokines including TNF-*α* and IL-6 (Habtemariam, 2020; Cui H. et al., 2018; Cui H. X. et al., 2018).

#### 4.2.4 Intervening in the microbiota–cancer axis: implications for colorectal cancer

Cluster analysis of the WOSCC keyword network reveals the presence of cluster #2, labeled “colon cancer growth” (Figure 6). Concurrently, “colorectal cancer” exhibits sustained burst activity beginning in 2021 (Figure 8), highlighting this area as a growing research focus. Key co-cited references, including Zhang W et al. (2019) and Sun Q et al. (2022), provide essential evidence for understanding the underlying mechanisms. Berberine exerts a chemopreventive effect against colorectal cancer (CRC), providing compelling evidence for the “gut microbiota-cancer axis”. Mechanistically, its action is multifaceted: (1) it directly exhibits anti-tumor activity by inhibiting cancer cell proliferation, inducing apoptosis, and suppressing oncogenic pathways such as Wnt/*β*-catenin (Lin et al., 1999; Zhang et al., 2024; Sun et al., 2022; Wang et al., 2018; Gao et al., 2017); (2) it indirectly modulates the gut microbiota ecosystem, correcting dysbiosis, reducing the production of pro-carcinogenic secondary bile acids like deoxycholic acid (DCA), and increasing the levels of anti-inflammatory and anti-cancer SCFAs, thereby improving the tumor microenvironment(Zhang et al., 2024; Sun et al., 2022; Wang et al., 2018); and (3) it enhances the intestinal barrier function, preventing bacterial translocation and the entry of microbial metabolites, which reduces chronic inflammation-driven carcinogenesis.

#### 4.2.5 Gut–brain dialog in neuropsychiatric disorders: Parkinson’s disease and depression as paradigms

In the CNKI database, “depression” emerged as a burst keyword starting in 2021 (Figure 7). Within the WOSCC dataset, the presence of burst terms such as “activated protein kinase” and “microbiota” (Figure 8) further points to a potential link with neuroimmune pathways. Highly cited works in the co-citation network, including Wang Y (2017) and Ma et al. (2022) (Table 3), exemplify this emerging research paradigm centered on the gut–brain axis. Emerging evidence indicates that BBR exerts its effects via the gut-brain axis. Parkinson’s Disease (PD): Gut microbiota metabolizes BBR into dihydroberberine, which promotes microbial synthesis of tetrahydrobiopterin (BH4). BH4 activates tyrosine hydroxylase (TH), ultimately enhancing the production of microbiota-derived L-dopa. Following its absorption, L-dopa is transported to the brain, converted into dopamine, and alleviates PD symptoms (Wang et al., 2021; Ma et al., 2022). Depression: BBR likely confers antidepressant effects through multiple gut-brain pathways: modulating gut microbiota composition, increasing levels of SCFAs, which influence neurotransmitter synthesis and brain function, and regulating levels of monoamine neurotransmitters (e.g., serotonin [5-HT], dopamine [DA]) as well as brain-derived neurotrophic factor (BDNF) expression (He, 2023). Together, these findings align with the emergence of “depression” as a burst term, positioning the gut–brain axis as a key growth area for future research.

#### 4.2.6 Combatting atherosclerosis: microbial and metabolic mechanisms

In line with this, Zhu et al. (2018) reported that berberine treatment markedly enriched *Akkermansia* abundance in the gut of HFD-fed Apoe−/− mice, an effect accompanied by reduced atherosclerotic lesions, lower metabolic endotoxemia, and improved intestinal barrier integrity as evidenced by increased tight junction protein expression and enhanced colonic mucus thickness. These observations indicate that despite its limited oral bioavailability, berberine exerts anti-atherosclerotic effects at least in part through microbiota modulation (Table 3)—a notion further supported by the appearance of “activation” and related terms among the burst keywords, pointing to the involvement of specific metabolic pathways. BBR and its gut microbial metabolites (e.g., dihydroberberine) effectively suppress the activity of the gut microbial choline/carnitine TMA-lyase (CutC/D), thereby reducing the production of TMA and its oxidized metabolite, TMAO. As TMAO is an independent risk factor for atherosclerosis, inhibition of this pathway represents one of the key mechanisms underlying its cardiovascular protection (Wang et al., 2021; Zhu et al., 2018; Shi et al., 2018).

#### 4.2.7 Organ-protective effects beyond the gut: the gut–liver and gut–lung axes

The sustained presence of “bile acids” among keywords, together with liver-focused co-cited studies such as Tian Y (2019), points to the emerging relevance of the gut–liver axis—even though this specific term is not flagged as a burst keyword. Studies suggest that BBR may modulate the gut microbiota and its metabolites (e.g., SCFAs, LPS) via the gut-X axis (such as the gut-lung axis and gut-liver axis), thereby indirectly influencing pathological processes (e.g., fibrosis) in distal organs (such as the lung and liver) (Guan et al., 2018; Liu et al., 2015).

### 4.3 Challenges and future perspectives

Despite the promising therapeutic potential revealed by research into berberine -gut microbiota interactions, several major challenges persist. These include BBR’s extremely low oral bioavailability (<1%), the impact of inter-individual microbiota variations on its efficacy, insufficient mechanistic understanding of the complex interactions, and a relative scarcity of high-quality clinical evidence.

The foremost challenge is BBR’s low oral bioavailability (<1%) (Han et al., 2021; Chen et al., 2011), a major clinical bottleneck. Consequently, developing novel delivery systems (e.g., nano-formulations (Gao, 2024), biofilm-based delivery) to enhance its local intestinal concentration and/or systemic absorption efficiency represents a critical research priority. Furthermore, the depth and complexity of the mechanisms require urgent attention: significant inter-individual microbiota differences lead to variable BBR responses, necessitating deeper investigation into the specific metabolic pathways, key enzymes, and regulatory mechanisms by which distinct gut bacterial species process BBR. Precise causal links and signaling mechanisms connecting shifts in microbiota structure to host physiological/pathological phenotypes (metabolic, immune, neurological), such as specific SCFA-receptor, bile acid-receptor, and microbial molecular pattern-host PRR interactions, remain inadequately defined, as do the intricate details of BBR’s modulation of cross-organ axes (gut-brain, gut-liver, gut-lung). Moreover, clinical translation is hampered by insufficient evidence; current mechanistic insights derive predominantly from animal models, highlighting a critical need for robust human clinical trials, particularly large-scale RCTs, to validate the clinical efficacy, safety, and optimal dosing regimens of microbiota-mediated BBR interventions for specific diseases (e.g., CRC, UC, depression). Additionally, future research must fully leverage and integrate advanced technologies and methodologies, including multi-omics approaches (metagenomics, metatranscriptomics, metabolomics, proteomics), germ-free animal models, microbiota transplantation studies, and advanced imaging techniques, to dissect the complex microbiota-host interaction network across multiple dimensions and levels. Finally, developing personalized intervention strategies based on host genotype and baseline microbiota features is essential to enhance treatment predictability and precision.

Future research should therefore prioritize: (1) the development of highly efficient novel delivery systems; (2) deep integration of multi-omics technologies and advanced models to elucidate the precise pathways of microbial BBR metabolism and microbiota-host signaling transduction mechanisms; (3) rigorous clinical trial design and implementation are needed to validate berberine’s clinical efficacy in specific diseases, including emerging therapeutic targets like CRC, UC, and depression.; (4) the exploration of precision intervention strategies guided by individual microbiota characteristics.

## 5 Conclusion

This study employed bibliometric methods (CiteSpace) to conduct a systematic visual analysis of the research field concerning berberine-gut microbiota interactions. Through an in – depth examination of 426 Chinese and English literature pieces, it clearly revealed the rapid development trend of this field, the research force distribution pattern dominated by China, and the collaborative networks among core institutions and authors. Literature retrieval was based on the WOSCC and CNKI databases, which cover the main journals and dissertations in this field and can well reflect the core research context. However, it should be noted that as some literature exclusive to databases like Scopus and PubMed (e.g., Wu C, 2021 from PubMed) and not included in the above – mentioned databases was not considered, there might be certain selection bias in the analysis. Despite these limitations, the analysis results of this study still offer a reference basis for sorting out the knowledge structure, analyzing research trends, identifying collaboration opportunities, and planning future key directions in the field of berberine – gut microbiota interactions.

## Data Availability

The original contributions presented in the study are included in the article/supplementary material, further inquiries can be directed to the corresponding authors.
